# Lack of evidence for association of primary sclerosing cholangitis and primary biliary cirrhosis with risk alleles for Crohn's disease in Polish patients

**DOI:** 10.1186/1471-2350-9-81

**Published:** 2008-08-21

**Authors:** Pawel Gaj, Andrzej Habior, Michal Mikula, Jerzy Ostrowski

**Affiliations:** 1 Department of Gastroenterology and Hepatology, Medical Center for Postgraduate Education at the Maria Skłodowska-Curie Memorial Cancer Center, Institute of Oncology, Warsaw, Poland

## Abstract

**Background:**

Numerous papers have addressed the association of mutations and polymorphisms of susceptibility genes with autoimmune inflammatory disorders. We investigated whether polymorphisms that confer susceptibility to Crohn's disease could be classified also as predisposing factors for the development of primary sclerosing cholangitis and primary biliary cirrhosis in Polish patients.

**Methods:**

The study included 60 patients with CD, 77 patients with PSC, of which 61 exhibited IBD (40 UC, 8 CD, and 13 indeterminate colitis), and 144 patients with PBC. All the patients were screened against Crohn's disease associating genetic polymorphisms.

The polymorphisms were chosen according to previously confirmed evidence for association with Crohn's disease, including Pro268Ser, Arg702Trp, Gly908Arg and 1007fs in *NOD2/CARD15*, Leu503Phe/-207G>C in *SLC22A4/OCTN1*/*SLC22A5/OCTN2*, Arg30Gln in *DLG5*, Thr300Ala in *ATG16L1*, and Arg381Gln, His3Gln and exon-3'UTR in *IL23R*. Genotyping was carried out using TaqMan SNP genotyping assays.

**Results:**

We confirmed a strong association between three *NOD2/CARD15 *gene variants (Pro268Ser, OR = 2.52, 95% CI = 1.34 – 4.75); (Arg702Trp, OR = 6.65, 95% CI = 1.99 – 22.17); (1007fs, OR = 9.59, 95% CI = 3.94 – 23.29), and a weak association between both the protective *OCTN1/OCTN2 *CC haplotype (OR = 0.28, 95% CI = 0.08 – 0.94), and a variant of *ATG16L1 *gene (Thr300Ala, OR = 0.468, 95% CI = 0.24 – 0.90) with Crohn's disease. In contrast, none of the polymorphisms exhibited association with susceptibility to primary sclerosing cholangitis and primary biliary cirrhosis, including a group of primary sclerosing cholangitis patients with concurrent IBD.

**Conclusion:**

Although the clinical data indicate non-random co-occurrence of inflammatory bowel disease and primary sclerosing cholangitis, consistently with the previously published studies, no genetic association was found between the genetic variants predisposing to Crohn's disease and hepatobiliary autoimmune disorders. However, since estimation of genetic variant disproportion is limited by sample size, these negative results may also indicate that eventually shared genetic predispositions are too little to be captured by small patient groups.

## Background

Identification of the mechanisms of autoimmune inflammatory disease development in the gastrointestinal tract is an emerging topic of research. Inflammatory bowel disease (IBD) is thought to result from immune-mediated tissue injury, primed by the enteric microflora, in genetically predisposed subjects. IBD phenotypically occurs in the form of Crohn's disease (CD) or ulcerative colitis (UC). In these disorders, chronic mucosal inflammation results from inappropriate and overreactive mucosal response to intestinal bacteria followed by activation of inflammatory cells and production of inflammatory mediators [1]. Primary sclerosing cholangitis (PSC) is a chronic inflammatory disease of the hepatic bile ducts that likely develops as a result of an inappropriate immune mediated process [2]. Nearly 80% of northern European PSC patients also have concomitant IBD, mostly in the form of UC [3]. Primary biliary cirrhosis (PBC) is another autoimmune chronic cholestatic liver disorder that may also develop as a result of an abnormal immune response to stimulating environmental or infectious agents [4].

As in other autoimmune diseases, the specific alleles of the human leukocyte antigen (HLA) complex represent genes associated with susceptibility to PSC [5-7], PBC [8,9] and IBD [10]. It has been reported that the genetic predisposition to CD is caused by alterations in several genes, including *NOD2/CARD15, OCTN1*, *OCTN2*, *DLG*, *ATG16L1 *and *IL23R *[11-14]. Several other non-HLA genes are also involved in PCS and PBC disease susceptibility [7,15].

According to our current understanding of CD, PSC and PBC are complex diseases in which the genetic determinants contributing to disease susceptibility interact with environmental factors. However, the mechanisms underlying these interactions remain unclear. Considering high frequency at which IBD and PSC are diagnosed together [3], close spatial proximity of affected organs, and the fact that common immune mediated processes are likely involved in development of these diseases [16], searching for common risk alleles for IBD and autoimmune cholestatic liver disorders seems to be relevant

Most of the susceptibility genes are considered to not be directly involved in disease pathogenesis, but rather act as environmental response modifiers. Given their subtle individual impact on disease susceptibility, independently they may have little to no effect on disease progression and likely act in collaboration with other factors to promote disease development. Moreover, disease genetic markers vary among the populations studied, despite the phenotypic similarity of disease. Thus, analyzing the genetic background of disease is an important challenge, and several studies have been performed with the purpose of finding similarities in the molecular mechanism of development of different inflammatory disorders of the alimentary tract. The aim of this study was to investigate whether polymorphisms of CD susceptibility genes are predisposing factors to the development of PSC and PBC in a group of Polish patients.

## Methods

### Patients

The study included 60 patients with CD (28 men, range of 22 – 78 years old; median 39), 77 patients with PSC (45 men, range of 20 – 68 years old; median 33), of which 61 exhibited IBD as confirmed by endoscopy and histology (40 UC, 8 CD, and 13 indeterminate colitis), and 144 patients with PBC (8 men, range of 40 – 72 years old; median 58). All patients were diagnosed at the Department of Gastroenterology and Hepatology, Medical Center for Postgraduate Education and Cancer Center, Warsaw, Poland. Diagnosis of CD was based on generally accepted clinical, endoscopical and histological criteria. The diagnosis of PSC was based on characteristic biochemical and radiological features (irregularity of the intrahepatic and extrahepatic bile ducts in endoscopic retrograde or magnetic resonance cholangiopancreatography). PBC was diagnosed based on generally accepted clinical, biochemical, serological (antimitochondrial antibody positivity by indirect immunofluorescence) and histological findings [17].

The control group consisted of 139 healthy volunteers (74 men, range of 21 – 66 years old, median 32) recruited from hospital staff, medical students, and healthy subjects selected for screening colonoscopy. All patients and controls were Polish Caucasians. The study was approved by the local ethics committee (Medical Center for Postgraduate Education and Cancer Center, Warsaw, Poland), and all the participants provided appropriate consent. The study protocol conforms to the ethical guidelines of the 1975 Declaration of Helsinki.

### Genotyping

Genomic DNA was extracted from whole blood treated with EDTA using the NucleoSpin Blood Quick Pure kit (Macherey-Nagel, Germany), following the manufacturer's protocol.

The selected polymorphisms of genes with previously confirmed evidence for association with CD were as follows: *NOD2/CARD15*: rs2066842 (Pro268Ser), rs2066844 (Arg702Trp), rs2066845 (Gly908Arg), rs5743293 (1007fs); in the *SLC22A4/OCTN1*/*SLC22A5/OCTN2 *genes: rs1050152 (Leu503Phe)/rs2631367 (-207G>C); in the *DLG5 *gene: rs1248696 (Arg30Gln); in the *ATG16L1 *gene: rs2241880 (Thr300Ala); and in the *IL23R *gene: rs11209026 (Arg381Gln), rs1884444 (His3Gln), rs10889677 (exon-3'UTR).

Genotyping was carried out using TaqMan SNP Genotyping Assays (Applied Biosystems; Foster City, CA). The PCR reactions were performed in 96-well plates on the ABI PRISM 7000 Sequence Detection System (SDS) (Applied Biosystems; Foster City, CA). Each well contained 20 ng genomic DNA, 1.25 μL TaqMan SNP Genotyping Assay (probes and primers mix), 12.5 μL TaqMan Universal PCR Master Mix, No AmpErase UNG (Applied Biosystems; Foster City, CA), and 9.25 μL water. Two non-template-control wells were included on each plate. After DNA amplification (95°C for 10 min, followed by 40 cycles of 92°C for 15 sec and 60°C for 1 min), fluorescence was acquired and analyzed for allelic discrimination using the ABI Prism 7000 SDS Software.

### Statistical analyses

The frequency distribution of alleles, genotypes, and haplotypes was compared using standard or Yates corrected χ^2 ^test and Fisher's exact test when appropriate. Statistical significance threshold level was Bonferroni corrected for multiple hypothesis testing, according to the number of genes tested. The p-value significance threshold level was defined as 0.008. For the Fisher's exact tests, the STATISTICA software was used. ORs with 95% confidence intervals (95% CI) were calculated using a calculator available at . LD analysis and calculation of the Hardy-Weinberg equilibrium were performed using the Haploview v3.2 software, available at . Statistical power analyses were done using G*power's (ver. 3.0.10) [18] post-hoc procedure for the Fisher's exact test.

## Results and Discussion

Allelic distributions for all polymorphisms studied in the patient and control groups are shown in Table 1, while genotype counts for each of the studied groups are presented in Additional file 1 [see Additional file 1]. In the control group, the genotype distributions for all polymorphisms were in Hardy-Weinberg equilibrium (p > 0.05).

**Table 1:** Allelic distribution in patients and healthy controls for all polymorphisms

Polymorphism	Genotype	CD	PSC	PBC	Controls
NOD2/CARD15 Pro268Ser	C/C	0.33	0.48	0.58	0.56
	C/T	0.40	0.45	0.36	0,41
	T/T	0.27	0.06	0.06	0.03
NOD2/CARD15 Arg702Trp	C/C	0.83	0.92	0.93	0.97
	C/T	0.17	0.08	0.07	0.03
	T/T	0.00	0.00	0.00	0.00
NOD2/CARD15 Gly908Arg	G/G	0.95	0.96	0.97	0.94
	G/C	0.05	0.04	0.03	0.06
	C/C	0.00	0.00	0.00	0.00
NOD2/CARD15 1007fs	-/-	0.63	0.91	0.92	0.94
	-/C	0.27	0.08	0.08	0.06
	C/C	0.10	0.01	0.00	0.00
OCTN1 Leu503Phe	C/C	0.27	0.35	0.37	0.37
	C/T	0.50	0.52	0.43	0.46
	T/T	0.23	0.13	0.20	0.17
OCTN2-207G>C	G/G	0.25	0.28	0.30	0.27
	G/C	0.47	0.54	0.43	0.46
	C/C	0.27	0.18	0.27	0.27
DLG5 Arg30Gln	C/C	0.80	0.77	0.82	0.80
	C/T	0.18	0.21	0.17	0.19
	T/T	0.02	0.03	0.01	0.01
IL23R Arg381Gln	G/G	0.97	0.90	0.92	0.95
	G/A	0.03	0.09	0.08	0.05
	A/A	0.00	0.01	0.00	0.00
IL23R His3Gln	C/C	0.19	0.26	0.23	0.30
	C/A	0.59	0.57	0.54	0.49
	A/A	0,22	0.17	0.23	0.22
IL23R exon-3'UTR	C/C	0.40	0.53	0.51	0.49
	C/A	0.52	0.41	0.40	0.42
	A/A	0.08	0.07	0.08	0.09
ATG16L1 Thr300Ala	C/C	0.39	0.29	0.25	0.23
	C/T	0.42	0.51	0.52	0.50
	T/T	0.19	0.20	0.23	0.27

The *Caspase Recruitment Activation Domain 15 *(*NOD2/CARD15*) gene is located in the proximal region of chromosome 16 (16q12) and encodes the nucleotide-binding oligomerization domain protein 2 (NOD2), which is a cytosolic receptor for a bacterial peptidoglycan response pathway [19]. Mutations in NOD2 result in immune system dysfunctions [11,20] and are considered causative genetic factors in the development of CD. *NOD2/CARD15 *mutations are also associated with susceptibility to other granulomatous inflammatory disorders, such as early-onset sarcoidosis and Blau syndrome [21,22].

Three of the four *NOD2/CARD15 *variants tested (rs2066842, rs2066844, rs5743293) were strongly associated with CD (Table 2). In particular, a strong association was observed with Pro268Ser and 1007fs SNPs with odds ratio (OR) values over 10. Increased values of OR for homozygous risk genotypes confirm previously described codominance of these variants. The observed allele distribution was similar to those reported in other Caucasian CD patient groups [23-25].

**Table 2:** Statistical analyses for significant genotype associations

Patient group	SNP		Genotype	OR	95% CI	p-value	Stat. Power
Crohn's disease	NOD2/CARD15	Pro268Ser	C/T+T/T T/T	2.52 12.18	1.34 – 4.75 3.86 – 38.37	0.005 0.0000003	0.59 0.98
	NOD2/CARD15	Arg702Trp	C/T+T/T T/T	6.65 n/a	1.99 – 22.17 n/a	0.0013 n/a	0.71 n/a
	NOD2/CARD15	1007fs	-/C+C/C C/C	9.59 >16	3.94 – 23.29 ---	0.00000002 0.0006	0.99 0.86
	ATG16L1	Thr300Ala	T/C+T/T T/T	0.468 0.632	0.24 – 0.90 0.30 – 1.34	0.022 0.231	0.32 0.05

*The autophagy-related 16-like 1 *(*ATG16L1*) gene encodes the protein component of the autophagosome pathway of intracellular bacteria processing. The T300A variant (rs2241880) was previously considered to be a risk factor for CD development [26,27]. In our CD patients, however, this variant demonstrated only a weak associaf2tion with CD, as the p-value (= 0.022) did not pass the Bonferroni corrected significance threshold level of 0.008.

The organic cation transporters from the family of solute carrier protein (*SLC22A4/OCTN1 *and *SLC22A5/OCTN2*) genes are located on chromosome 5 (5q31) within the IBD5 region [28]. The 1672C/T (Leu503Phe) missense variant of *SLC22A4/OCTN1 *(rs1050152) potentially alters the carnitine adsorption and the exchange of other positively charged compounds between the cell and extracellular matrix [29]. Also included in this study is the G-to-C transversion in the *SLC22A5/OCTN2 *promoter (rs2631367), which is thought to affect a heat shock transcription factor binding element [30]. The variants of both genes were previously found in linkage disequilibrium, enabling the selection of a two-allele CD risk haplotype [28,31]. This finding, however, has not been confirmed by other studies [29].

As shown in Table 3, there was a weak association between the protective *OCTN1/OCTN2 *CC haplotype and CD (OR = 0.28; CI = 0.08 – 0.94; p = 0.0298). A strong linkage disequilibrium was found between the *NOD2/CARD15 *Pro268Ser loci and three other studied loci of *NOD2/CARD15*. Analyses of reconstructed haplotypes for each patient group showed that the odds of TC haplotype of Pro268Ser and 1007fs loci is approximately ten times higher (OR = 10.26; CI = 4.52 – 23.29; p = 0.0000000000671) among patients with CD compared to healthy controls.

**Table 3:** Statistical analyses using Haploview software (ver. 3.2) for significant haplotype associations.

Patient group	SNP	Haplotype	OR	95% CI	p-value
Crohn's disease	NOD2/CARD15 Pro268Ser/Arg702Trp	TC	2.25	1.41 – 3.58	0.0005
	NOD2/CARD15 Pro268Ser/Gly908Arg	TG	3.01	1.89 – 4.78	0.000002
	NOD2/CARD15 Pro268Ser/1007fs	TC	10.26	4.52 – 23.29	0.0000000000671
	OCTN1/OCTN2	CC	0.28	0.08 – 0.94	0.0298

The *interleukine-23 receptor *(*IL23R*) gene is located on chromosome region 1p31, and encodes a subunit of the IL23 receptor. IL23 and IL6 activate the STAT3 transcription factor which in turn leads to differentiation of CD4+ helper T cells into Th17 cells that function in driving autoimmune inflammation by producing pro-inflammatory cytokines, such as IL-17A, IL-17F and IL-22 [13,32-35]. Genetic variants of *IL23R *were identified as disease-associated factors in chronic inflammatory disorders, such as IBD [12], psoriasis, T-cell-mediated inflammatory disease of the skin [36], and ankylosing spondylitis [37], indicating the involvement of the IL23/IL23R pathway in the etiology of various autoimmune-related disorders. Several genetic non-coding variants of this gene demonstrated independent risk association with CD, while the rare variant in the coding region of *IL23R *(1142G/A; Arg381Gln; rs11209026) has been regarded as a CD protective variant [12,13].

We did not find any association of CD not only with Arg381Gln, but also two other more frequent variants of *IL23R*. The frequency of the G/A genotype of rs11209026 SNP was 3.3% in CD (2 of 60) compared to 5.8% (8 of 139) in controls, and only one of the patients and controls was homozygous for the AA genotype (not significant). Thus, although this rare variant was found at a similar proportion to that described from the genome-wide scan-based population studies (2.5–3.0% *versus *6.2–6.8%; [13,38]), our small group of CD patients (N = 60) was insufficient to reach statistical significance for this disproportion.

The *Drosophila *disc large homolog 5 (*DLG5*) gene, mapped to chromosome 10 (10q23), is a member of the membrane-associated guanylate kinase gene family [10,39] and encodes a protein involved in maintaining the correct shape, polarity and growth of cells. The functional variant (113G/A, Arg30Gln, rs1248696) has been proposed to potentially lead to serious abnormalities in both the structure and function of cells. Although the first study of Stoll *et al. *[40] reported an association between genetic variants in *DLG5 *and IBD, the more recent meta-analysis of published data on Arg30Gln question this association [10]. Our study did not show any association between the Arg30Gln variant of *DLG5 *and CD.

In contrast to CD patients, none of the polymorphisms showed association with PSC and PBC, two disorders of autoimmune etiology. Furthermore, no significant associations were detected in PSC patients with concurrent IBD. These negative results are consistent with the recently published studies in Scandinavian PSC patients, the largest PSC population in which genotyping has been performed [2], and in a group of Hungarian and Polish patients with PBC [41].

The present study confirms a strong association of *NOD2/CARD15 *gene variants, and to a lesser extent the coding *ATG16L1 *variant and the *OCTN1/OCTN2 *haplotype, with CD in a relatively small Polish Caucasian patient group. There was no evidence for significant association between variants of *IL23R *or *DLG5 *and CD. However, because small sample size significantly limits estimation of genetic variant disproportion, especially with a low prevalence of allelic frequency, whether the result represents a true negative association or a false negative finding is somewhat disputable, mainly due to insufficient power of statistical tests [7]. In fact, calculation of the statistical testing power reached decent values only for the three main *NOD2/CARD15 *variants (Table 4). Therefore, considering SNP frequencies, small patient groups and measured effect size, our studies might not be able to confirm existing weak and even moderate disease associations.

**Table 4:** Power of genotype association statistics given as 1-β error probability. The β represents the probability of falsely accepting H0 hypothesis (lack of association) when in fact H1 hypothesis (association) is true.

Polymorphism	Genet. model	CD	PSC	PBC
NOD2/CARD15 Pro268Ser	dominant	0.59	0.04	<0.01
	recessive	0.98	0.03	0.04
NOD2/CARD15 Arg702Trp	dominant	0.71	0.11	0.08
	recessive	n/a	<0.01	n/a
NOD2/CARD15 Gly908Arg	dominant	<0.01	<0.01	0.05
	recessive	n/a	n/a	n/a
NOD2/CARD15 1007fs	dominant	0.99	0.02	0.01
	recessive	0.86	<0.01	n/a
OCTN1 Leu503Phe	dominant	0.08	<0.01	<0.01
	recessive	0.04	0.02	0.02
OCTN2-207G>C	dominant	<0.01	<0.01	0.01
	recessive	<0.01	0.09	<0.01
DLG5 Arg30Gln	dominant	<0.01	0.02	0.01
	recessive	<0.01	0.02	<0.01
IL23R Arg381Gln	dominant	<0.01	0.08	0.03
	recessive	<0.01	<0.01	n/a
IL23R His3Gln	dominant	0.09	0.01	0.06
	recessive	<0.01	0.02	<0.01
IL23R exon-3'UTR	dominant	0.05	0.01	0.01
	recessive	<0.01	0.01	<0.01
ATG16L1 Thr300Ala	dominant	0.32	0.04	0.01
	recessive	0.05	0.04	0.02

## Conclusion

Although inappropriate immune mediated processes are also related with PSC and PBC, this study and previously published [2,41] studies have not identified common allelic variants. In IBD, risk variants of genes encoding proteins that are involved in signalling pathways activated by bacterial products have been described. Consequently, IBD is thought to result from dysfunction of the mucosal immune response triggered by intestinal bacteria, and defects in the early immune response may play a role in the pathogenesis of CD [1,42,43]. However, this hypothesis is based on statistical considerations rather than functional studies. It is noteworthy that only some CD patients carry mutated *NOD2/CARD15 *and/or other risk gene variants, and therefore, mechanisms for CD development likely are not simply related to mutations within described risk genes. Furthermore, the genetic background of CD possibly is based on more causative genetic factors than studied thus far, and each of them independently may have a relatively weak impact on disease development.

Such high complexity of disease background requires mapping of genetic predisposition using genome-wide and large-scale studies in well-defined populations. Despite the fact that these kind of studies are still relatively expensive, they are likely the most cost effective giving real capacity to provide comprehensive insight into disease development mechanisms.

Even though familial aggregation is observed in many complex diseases, including IBD, PSC and PBC, genetic factors in complex diseases should be considered as "risk-factor genes" rather than genes responsible for the development of a particular disease. Their presence is not synonymous with the development of the disease. Thus, functional studies should be initiated in order to clarify the contribution of genetic background in development of these diseases.

## Competing interests

The authors declare that they have no competing interests.

## Authors' contributions

PG participated in preparation of the study design, carried out the molecular assays as well as performed statistical analyses and helped to draft the manuscript. AH provided diagnoses for the patients and enrolled patients eligible for the study. MM participated in preparation of the study design. JO conceived of the study, and participated in its design and coordination and drafted the manuscript. All authors read and approved the final manuscript.

## Availability & requirements





## Pre-publication history

The pre-publication history for this paper can be accessed here:



## Supplementary Material

Additional file 1 genotype counts. genotype counts for each of the studied groups Click here for file
